# Does self-compassion moderate the relationship between stigma, pain and psychological well-being among people living with sickle cell disease in Ghana?

**DOI:** 10.1371/journal.pmen.0000556

**Published:** 2026-02-23

**Authors:** De-Graft Nana Agyei, Benjamin Amponsah, Annabella Osei-Tutu

**Affiliations:** 1 Department of Psychology, Virginia Commonwealth University, Richmond, Virginia, United States of America; 2 Department of Psychology, University of Ghana, Accra, Ghana; PLOS: Public Library of Science, UNITED STATES OF AMERICA

## Abstract

Sickle cell disease (SCD), a chronic genetic disorder, is associated with severe pain, stigma, and psychological distress, particularly in low-resource settings like Ghana. In this study, we used convenient and purposive sampling to explore the role of self-compassion in relation to pain, stigma, specifically SCD-related stigma encompassing social exclusion, internalized stigma, disclosure concerns, anticipated discrimination, and psychological well-being among people living with SCD. We recruited 138 adults (46, [33.3%] males, 92, [66.7%] females; mean age = 30.99, SD = 8.44) with SCD from two Non-Governmental Organizations (NGO) in Accra, Ghana. Self-compassion had significant negative correlations with pain (r = -.21, p <.01) and stigma (r = -.37, p <.01), and a positive correlation with psychological well-being (r =.53, p <.01). It also independently predicted better psychological well-being (B = 1.93, p <.001), emphasizing its supporting role in well-being. However, self-compassion did not moderate the effects of pain (B =.02, p =.512) or stigma (B = -.02, p =.852) on psychological well-being. Future research should explore the implementation of interventional studies focused on enhancing self-compassion to examine its causal effects on pain management, stigma reduction, and improvements in psychological well-being among individuals living with sickle cell disease.

## Introduction

Sickle cell disease is a genetic blood disorder that affects hemoglobin, the protein in red blood cells responsible for carrying oxygen throughout the body [1]. It has been identified as the most common life-threatening genetic illness among persons of African ancestry, as well as those from the Middle East, Mediterranean basin and the Indian subcontinent [2]. It is a huge public health concern in Africa, with over 200,000 newborns being affected each year [3]. Some studies have shown that around 80% of people with sickle cell disease are born in Sub-Saharan Africa [4,5]. In Ghana, approximately 15,000 newborns are affected by SCD annually, with an estimated prevalence of 1 in every 50 births [6]. For instance, among 2,400 babies born on New Year’s Day in 2018, a notable proportion were diagnosed with SCD, often without prior awareness by their parents [6].

Sickle cell disease is clinically characterized by a triad of chronic hemolytic anemia, recurrent vaso-occlusive crisis (VOC), and progressive organ damage, which collectively reduce life expectancy [7]. These physical symptoms are often accompanied by psychological consequences, including diminished quality of life [8], reduced psychological well-being [9], and experiences of stigma [10]. It is a profoundly serious condition that causes significant suffering and may lead to death [11]. SCD impacts on all areas of an individuals’ life, often resulting in painful crises. As a consequence of these pain crises, individuals living with the condition frequently experience discrimination and stigmatization. This stigma can come from the public, at times, from healthcare professionals, who may view patients as drug seekers [12]. Such perceptions can lead to delays in receiving medical care, particularly in emergency departments, where patients’ experiences may not be believed [12]. Moreover, the stigma associated with SCD can limit individuals in various aspects of life, including finding a partner and disclosing their condition to employers [13]. Overall, the challenges posed by SCD extend beyond physical health, affecting emotional well-being and social interactions. Previous research has shown that individuals living with SCD often experience lower psychosocial well-being due to chronic pain, frequent hospitalizations, and social stigma. Studies have documented elevated levels of anxiety, depression, and emotional distress in this population, particularly among those with limited access to psychosocial support [14,15]. Moreover, psychological well-being in SCD populations has been linked to coping strategies, social support and perceived discrimination [16]. Despite these findings, few studies have explored positive psychological constructs such as self-compassion in relation to well-being among individuals with SCD, especially in African contexts. This study addresses that gap by examining how self-compassion relates to pain, stigma, and psychological well-being in a Ghanaian sample.

Biomedics have made significant contributions to alleviating the pain associated with SCD by developing medications aimed at reducing or relieving this pain [17]. While these medications are beneficial for people living with the condition, they often come with associated side effects. The biopsychosocial model [18] suggests that treatment can be more holistic when considering the social aspects of the disease. Some studies recommend psychological interventions that can complement the medical treatment of sickle cell disease which in a way can help reduce the side effects of these medications, and patients’ dependence on the medications [14,19,20]. Research has shown that psychological interventions can be effective in helping individuals with SCD manage their suffering [21,22]. By combining psychological with biomedical treatments, a more comprehensive approach to care can be achieved, ultimately contributing to the well-being of individuals living with SCD.

Self-compassion, as defined by [23,24], refers to “being open to and moved by one’s own suffering, experiencing feelings of caring and kindness toward oneself, taking an understanding, nonjudgemental attitude toward one’s inadequacies and failures, and recognizing that one’s experience is part of the common human experience”. This construct aligns with the biopsychosocial model, emphasizing emotional resilience, self-care, and holistic well-being. It comprises three core components: self-kindness versus self-judgement, common humanity versus isolation, and mindfulness versus over-identification [23,25–27]. It is particularly relevant when evaluating the well-being of individuals living with chronic conditions, such as sickle cell disease. People who practice self-compassion often experience joy, contentment, and motivation, while also enjoying healthier relationships and better physical health. They additionally tend to experience fewer feelings of anxiety and depression [24]. Drawing a clue from self-compassion interventions, it may be helpful for individuals living with SCD. Self-compassion can be a valuable tool in managing pain and coping with the stigma they may encounter during debilitating episodes. Compassionate and empathetic care is crucial in ensuring that people living with SCD adhere to regular screenings and treatments necessary for managing their condition. It is essential to teach individuals, especially those with chronic conditions like SCD, to show themselves the same level of kindness, support, and empathy they would offer a friend going through a difficult time.

### Self-compassion, pain management and well-being

Self-compassion is established in numerous studies to help people manage their pains, especially chronic pain with psychological components. For instance, [28] explored whether a 12-week compassion-focused therapy program would help individuals experiencing chronic pain relieve them from their pains. They found that individuals enduring persistent pain with a significant psychological aspect experienced relief through Compassion Focused Therapy. This approach helped alleviate feelings of isolation, enhanced self-reassurance, introduced new coping mechanisms, and fostered a growing acceptance of the limitations imposed by their pain. A feasibility study by [29] assessed a minimally supervised online self-compassion program tailored for chronic pain. This program used selected elements from a well-known self-compassion psychoeducation website, adapting them to be relevant for pain management. The findings suggestedself-compassion approach could be beneficial for adults with chronic pain, particularly those with limited access to traditional psychological services or who prefer online interventions.

Self-compassion is increasingly recognized as pivotal in helping mental health and well-being. Some studies have shown that self-compassion is beneficial to both a healthy and unhealthy population and helps people relieve physical symptoms and increases their psychological well-being. [30] assessed the relationship between self-compassion, physical symptoms and psychological well-being in college students and they established that self-compassion is associated with less physical symptoms and increased psychological well-being. However, they found that when individuals engage in more self-judgment, they are less likely to engage in healthy behaviors and tend to affect their psychological well-being. A meta-analytic study by [31] examined 20 samples from 14 studies to evaluate the correlation between self-compassion and mental illness. They found that self-compassion leads to better mental health and fewer mental health issues. Similarly, [32] found out in their meta-analytic study that people who practice self-compassion have higher levels of cognitive and psychological well-being. Lastly, [33] found that self-compassion is negatively associated with rumination and psychological symptoms, suggesting that self-compassion may improve psychological health by mitigating the effects of rumination.

### Current study

Sickle cell disease has received considerable research attention in Ghana and across the African continent [3,34–36]. However, there is still limited understanding of the connections between pain, stigma, mental health and self-compassion. This gap necessitates research into the self-compassion variable in Ghana, which could provide insight for future studies. Understanding the role of self-compassion in managing SCD could lead to practical applications where practitioners use these techniques to help individuals manage their pain and the associated stigma. To effectively address issues of pain, stigma, and enhance psychological well-being, it is crucial to explore and investigate the psychosocial factors affecting these individuals. This exploration will serve as a roadmap to developing interventions using self-compassion to support people living with SCD.

In Ghana and across Africa, there is a paucity of research on self-compassion, particularly among individuals living with SCD. To the best of our knowledge, no studies to date have examined self-compassion in SCD populations*.* Thus, this study seeks to explore the role of self-compassion among individuals with SCD. Despite significant attention from researchers, and awareness creation from non-governmental organizations, and other agencies, many people with SCD still experience stigma.

In this study, we investigated the role of self-compassion in relation to pain, stigma, and psychological well-being among people living with SCD. Self-compassion has been shown to promote emotional resilience and reduce the impact of stressors on psychological outcomes [37,38]. In the context of chronic illness, individuals who practice self-compassion may be better equipped to cope with pain and stigma by responding with kindness, mindfulness, and a sense of shared humanity. Prior studies suggest that self-compassion can attenuate the psychological toll of adverse experiences, including physical symptoms and social rejection [39,40]. Based on this evidence, we hypothesized that self-compassion would moderate the relationship between pain and psychological well-being, and between stigma and psychological well-being, such that higher self-compassion would weaken the negative associations.Self-compassion will have an inverse relationship with pain and stigma.

We hypothesized that self-compassion would have an inverse relationship with pain and stigma.

## Method

### Study design

*This quantitative stud*y employed a self-reported cross-sectional design to purposively and conveniently sample participants from two non-governmental organizations (NGO) in Ghana.

### Participants

Our participants included 138 (46 males, 92 females) people who are members of the NGOs. The participants included people 18–65 years old. Most of our participants had attained tertiary education (n = 104), with most employed (n = 65). See Table 1. The sample size was determined using the rule of thumb suggested by [41], which recommends that the sample size (n) for regression analysis should be equal to or exceed 50 + 8m, where ‘m’ is the number of predictor variables. In this study, with three predictors (stigma, self-compassion, and pain), the minimum required sample size was 74. A sample size of 138 was considered statistically adequate, exceeding the minimum requirement.

**Table 1:** Sample characteristics of study sample (N = 138).

Characteristic	Frequency	Percentage (%)
**Sex**		
Male	46	33.3
Female	92	66.7
**Marital status**		
Single	101	73.2
Cohabiting	4	2.9
Married	31	22.5
Divorced	2	1.4
**Religion**		
Christian	124	89.9
Muslim	12	8.7
Other	2	1.4
**Educational level**		
Tertiary	104	75.4
Secondary	20	14.5
Basic	14	10.1
**Employment status**		
Employed	65	47.1
Unemployed	16	11.6
Self-employed	31	22.5
Student	26	18.8
**Sickle cell status**		
SS	97	70.3
AS	3	2.2
SC	38	27.5
**Age of sickle cell diagnosis**		
Newborn/infancy (birth-5 years)	68	49.3
Childhood (6–12 years)	42	30.4
Adolescent (13 years to 18 years)	14	10.1
Adulthood (19 and above)	14	10.1
**Frequency of pains**		
Rarely	29	21.0
Sometimes	56	40.6
Mostly	26	18.8
Frequently	27	19.6
**Mean Age (SD); min-max**	30.99 (8.44); 18-65	

Note. Hemoglobin SS- Sickle cell Anemia (SS); Sickle Cell Trait (AS); Hemoglobin SC Disease (SC).

### Procedure

#### Recruiting and sampling.

Ethical approval and clearance were obtained from the Ethics committee for Humanities of the University of Ghana (protocol number: ECH334/21-22). We also sought permission from two non-governmental sickle cell social support and advocacy groups in Ghana, Accra. These groups both provide social support, education and other counselling services for members living with sickle cell disease and caregivers. We specifically sampled individuals from NGOs because people living with sickle cell disease often face stigma from healthcare professionals during crises, who may label them as drug-seeking [42,43]*.* This stigma can negatively impact their mental health. However, research suggests that individuals who receive social support are more likely to cope effectively during crises [44,45]. Therefore, selecting participants from NGOs that provide social support, advocacy, and education enabled us to better explore the relationships between self-compassion, pain, stigma, and well-being in this population. Recruitment was conducted through WhatsApp group announcements and in-person support group meetings at the Korle-Bu teaching hospital and other meeting venues of these NGOs. Participants were informed about the study’s purpose, assured of confidentiality and anonymity, and reminded that participation was voluntary.

### Data collection

Data was collected using three different methods. First, participants completed paper-and-pencil questionnaires during support group meetings. These were self-administered, not interviewer-administered, and a total of 50 participants completed the survey in person. Second, a Google Forms link was shared via the WhatsApp platforms of both groups to reach participants who could not attend in-person meetings due to weather conditions; 83 participants completed the survey online. Third, for participants who did not have smartphones and were unable to attend in-person meetings but were still willing to participate, the first author administered the questionnaire verbally over the phone. Five participants completed the survey using this method. Participants mostly helped refer or reach their other members of the support groups. All participants were compensated with an amount of 20 Cedis for their time completing the questionnaires.

### Measures

#### Stigma (The Measure of Sickle Cell stigma).

Stigma was measured using the measure of sickle cell stigma adult scale [46] which is used to assess specific stigma associated with an adult living with sickle cell disease. The scale has 11 items, and a response range from 1 (completely false) to 6 (completely true). Our sample recorded a Cronbach alpha of.77. see Table 2 below.

**Table 2:** Means, standard deviations, range, skewness and kurtosis and reliability of psychological well-being, self-compassion, stigma, and pain scales.

*Variable*	*N*	*Minimum*	*Maximum*	*Mean*	*SD*	*Skewness*	*Kurtosis*	*Alpha* (α)
PWB	138	42	126	94.06	14.18	-.29	1.36	.71
SELF COM	138	8.50	28.00	19.54	3.30	-.19	.61	.73
PAIN	138	.00	50.00	24.46	11.94	-.18	-.65	.92
STIGMA	138	11.00	57.00	33.05	10.72	.19	-.57	.77

PWB=Psychological wellbeing; SELF COM= Self compassion.

#### Psychological Well Being (PWBS).

The psychological well-being scale [47,48] was used to measure psychological well-being. It consists of 18 items which assess the well-being of a person and has a response rate of 1 (strongly agree) to 7 (strongly disagree) with some items reversed scored. The Cronbach alpha for our sample was.71. *see* Table 2 above.

#### Pain (The short-form McGill Pain Questionnaire).

The short-form McGill Pain questionnaire [49] to test for pain was used to measure pain. The scale has 15 descriptors with 1 of them being sensory and the remaining 4 being affective. The first 11 items address the sensory dimension of pain, or how pain impacts the person. It is rated on an intensity scale of 0–3, severe is the highest rating (0 showing no pain, 1 being mild and 2 being moderate). The point values for responses to the 15 questions were summed. We had a Cronbach alpha of.92. *see* Table 2. above.

#### Trait Self-compassion (The self-compassion scale-short form).

The self-compassion scale-short form [50], which contains 12-item self-report to assess individuals’ level of compassion towards themselves. The items were reported on a 5-point response scale from 1 (almost never) to 5 (almost always) where participants rate how frequently they act in debilitating situations. Our sample had a Cronbach alpha of.73.

### Data analysis

Analysis was done using the IBM SPSS (v.29) which involved using descriptive statistics, checking for normality using measures of skewness and kurtosis and ensuring all other assumptions were met prior to the main analysis (See Table 2) We used the SPSS macro process (v4.1) [51] to analyze our moderated hypothesis. Skewness cut off values were ≤ ± 1 and kurtosis cut-off values were ≤ ± 2 acceptable for the parametric analysis [52–54]. To test the hypothesized associations among the variables, the moderator was entered into the W moderator space for further analysis after the DV was first entered into the Y category, the IV into the X category, and the hypothesis into the PROCESS macro in SPSS. We conducted correlations among study variables and can be found in Table 3 below.

**Table 3:** Intercorrelations among study variables [self-compassion, stigma, pain, and psychological wellbeing].

Variable	1	2	3	4
1. Psychological well being	–			
2. Self-compassion	.53* [.40,.64]	–		
3. Stigma	−.41* [−.54,−.27]	−.37* [−.50, −.21]	–	
4. Pain	−.48* [−.60, −.34]	−.22* [−.38, −.06]	.34* [.18,.48]	–

Note. N= 138; * p < 0.01 two-tailed. 95% CI [lower interval, Upper interval].

### Sensitivity analysis

A post-hoc sensitivity analysis was conducted using G*Power (version 3.1.9.7) to determine the minimum detectable effect size for the moderation (interaction) effect. With a sample size of 138, the analysis indicated that the smallest detectable effect size for the interaction term was f² = 0.058 (ΔR² ≈ 0.055), which is considered a small effect according to [55] benchmarks. This suggests the study was adequately powered to detect small moderation effects in the relationships between pain or stigma and psychological well-being by self-compassion.

## Results

A moderation analysis was conducted to examine whether self-compassion moderates the relationship between pain and psychological well-being as well as self-compassion moderates the relationship between stigma and psychological well-being. Prior to analyses, the independent and moderator variables were centered. Hayes’ [51] PROCESS macro version 4.2 (Model 1) was used to generate 5,000 bootstrapped confidence intervals of the conditional effect

The result showed that the regression model was statistically significant F (3,134) = 32.76, *p* <.01. The variance explained in psychological well-being was 42%. We observed that pain (B = -.45) and self-compassion (B = 1.93) made statistically significant contributions to explaining the variance in psychological wellbeing. However, we did not find a significant interaction between self-compassion and pain in psychological well-being (B =.02, t =.65, *p* =.512). Thus, self-compassion did not moderate the relationship between pain and psychological well-being. The summary of the results is presented in Table 4.

**Table 4:** Results of moderation analysis examine the moderating effect of self-compassion on the relationship between pain and psychological well-being.

Variables	Β	SE	*t*	*p*
Constant	94.19	.95 [92.31, 96.06]	99.34	.<.01
Pain	−.45	.08 [−.61, −.30]	−5.66	.<.01
Self-compassion	1.93	.29 [1.36, 2.50]	6.68	.<.01
Self-compassion * Pain	.02	.02 [−.03,.06]	.65	.512

Note. R^2^ =.42. a. dependent variable: psychological wellbeing; upper and lower confidence interval in parenthesis is that of B.

In the second hypothesis on whether self-compassion would moderate the relationship between stigma and psychological wellbeing, the regression model was statistically significant (F (3,134) = 23.09, *p* <.01). The variance explained in psychological wellbeing (34%). We observed a statistically significant contribution of stigma (B = -.34, *p* <.01) and self-compassion (B = 1.91) to explain the variance in psychological wellbeing. We also did not find a significant interaction between self-compassion and stigma (B =.03, t = -.19, p =.85), that is, self-compassion did not moderate the relationship between stigma and psychological wellbeing in our sample. The summary of the results can be found in Table 5.

**Table 5:** Results of moderation analysis examining the moderating effect of self-compassion on the relationship between stigma and psychological wellbeing.

Variables	Β	SE	*t*	*p*
Constant	93.99	1.06 [91.90, 96.08]	89.10	.<.01
Stigma	−.34	.10 [−.54, −.14]	−3.34	.001
Self-compassion	1.91	.33 [1.26, 2.55]	5.86	.<.01
Stigma*Self-compassion	−.01	.03 [−.06,.05]	−.19	.852

Note.R^2^ =.34; a. dependent variable = psychological wellbeing; upper and lower confidence interval in parenthesis is that of B.

## Discussion

Self-compassion was tested whether it could moderate the relationship between pain and psychological well-being among people living sickle cell disease as well as whether self-compassion would moderate the relationship between stigma and psychological well-being.

### Self-compassion, pain and psychological well-being

Self-compassion had an inverse relationship with pain. This suggests that a high level of self-compassion is likely to be associated with a lower level of pain. Self-compassion, which involves being kind to oneself and accepting self in challenging situations, can help calm the nervous system. This self-soothing can stimulate the release of neurotransmitters like serotonin, which, when imbalanced, can trigger or exacerbate pain and it can also stimulate the release of neurotransmitters like oxytocin which may have pain relieving effect [56]. Additionally, available evidence shows that social stressors can exacerbate pain [57], by triggering or rekindling the release of these neurotransmitters. Therefore, this mechanism may explain the significant negative relationship between self-compassion and pain. This aligns with a study by [57] who found that self-compassion has a significant negative relationship with pain catastrophizing among people living with persistent musculoskeletal pain.

Self-compassion was expected to moderate the relationship between pain and psychological well-being, serving as a buffer to reduce pain among people living with sickle cell disease and enhance their psychological well-being. However, in our study, self-compassion did not moderate this relationship. It is important to note that the mean self-compassion score in our sample was relatively low, suggesting that participants may not have internalized strong self-compassionate tendencies. This low baseline may have limited the potential for self-compassion to function as a moderator. In populations with higher levels of self-compassion, buffering effects may be more likely to emerge. These findings underscore the importance of culturally adapted interventions that can help cultivate self-compassion in ways that resonate with the lived experiences of individuals with SCD in Ghana.

In addition to the low baseline scores, the absence of prior exposure to self-compassion interventions may have further limited its moderating potential. Another explanation may lie in the cultural and contextual relevance of self-compassion. In Ghanaian settings, where communal coping, religious faith, and family support are often emphasized, self-compassion as an individual-focused construct may not operate in the same way as in Western populations. It is possible that participants rely more on external sources of support than internal self-soothing strategies, which could limit the moderating role of self-compassion. Additionally, the chronic and unpredictable nature of SCD pain may overwhelm personal coping resources, making it difficult for self-compassion to buffer psychological distress without structured guidance or therapeutic reinforcement.

Previous interventional studies have established that techniques like relaxation, when practiced regularly for about three months, can help reduce emotional and physiological pain [58,59]. This suggests that self-compassion could potentially moderate the relationship between pain and psychological well-being if individuals receive proper training or interventions. This further supports the idea that without structured interventions, self-compassion may not be sufficiently developed to exert a buffering effect, especially when assessed through self-report measures. Our finding is not isolated, a study by [60] found that self-compassion was not a significant independent predictor of pain intensity or pain unpleasantness.

### Self-compassion, stigma and psychological well-being

The relationship between self-compassion and stigma was also negative. Sickle cell diseases come with stressors that may limit social functioning. For instance, individuals living with sickle cell disease are likely to avoid certain social events or situations due to the condition or pain associated with the disease. They may miss social gatherings or work frequently because of their pain, which can lead to social stigma. These individuals might be perceived as unreliable or even as drug addicts due to their frequent medication intake. Our result suggests that people who score high on self-compassion are likely to score low on stigma. This can be explained by the fact that individuals who score high on self-compassion are likely to not be self-critical about themselves. They would accept themselves for who they are and tend to be mindful of their surroundings. Self-compassion may help individuals in stigmatizing situations. This aligns with previous study indicating that self-compassion can alleviate feelings of shame and criticism [61].

However, we did not find a significant moderating relationship between self-compassion, stigma and psychological well-being. That is, self-compassion did not buffer the relationship between stigma and psychological well-being. This could be accounted for by the fact that we used only self-report measures to assess this without conducting any interventional approach and identifying if self-compassion is a helpful tool as demonstrated by other previous works [61,62]. This aligns with [63] finding that self-compassion could not moderate the connection between experienced stigma and distress.

Although our moderation hypotheses were not supported, this study contributes to valuable insights into the psychosocial experiences of individuals living with SCD in Ghana. By documenting the relationships among pain, stigma, self-compassion, and psychological well-being, we provide a foundation for future research and intervention development in this under-studied population. These findings highlight the need for culturally grounded approaches that address both emotional and social dimension of SCD, and they open the door for further exploration of positive psychological constructs in African health contexts.

### Limitation of study

Our study is not without limitations. The cross-sectional design employed in this study precluded making causal inferences. The results interpretation and discussion were based on prior theory and literature; That said, this study offers much insight on correlates of self-compassion among the sickle cell population in Ghana. A key limitation of this study is the recruitment of participants exclusively through NGO meetings (even though most of these participants do regular check-ups at the hospital). This strategy may have inadvertently excluded individuals who are less connected to support networks or who face greater barriers to engagement, such as those experiencing more severe stigma or limited mobility. As such, the sample may reflect individuals who are relatively more proactive in managing their condition. Future research should consider complementary recruitment strategies, including healthcare settings, to ensure broader representation of those most in need of psychosocial interventions.

Another limitation of this study is the selection of participants from a social support group. These individuals may differ from the broader population of individuals with SCD, particularly in terms of access to resources and regular support, which could influence their experiences and limit the generalizability of our findings. Social support can provide emotional and practical assistance, potentially buffering against the negative impacts of pain and stigma and enhancing psychological well-being. Additionally, the reliance on self-reported data, which may be subject to biases such as social desirability, particularly in reporting stigma. Lastly, we did not control confounding variables like SCD genotype, employment status, etc which may all impact outcomes like pain, stigma, and psychological well-being.

## Conclusion

Sickle cell disease is a chronic condition that comes with significant stressors, including recurrent pain episodes, stigma, and other challenges that can negatively impact psychological well-being. While self-compassion has shown to be a beneficial tool for managing stress and improving outcomes in other chronic diseases, the current study did not find a moderating effect of self-compassion on the relationship between pain, stigma, and well-being in individuals with sickle cell disease.

Despite the lack of a buffering effect, self-compassion may still offer important benefits for this population. By stimulating the release of neurotransmitters like serotonin and oxytocin, self-compassion can activate the parasympathetic nervous system and engage brain regions that promote relaxation, positive emotions, and resilience in the face of debilitating symptoms and stressors.

Additionally, the direct relationships found between self-compassion, pain, stigma, and well-being suggest that promoting self-compassion could have independent benefits for psychological health, regardless of its moderating capacity. However, the specific mechanisms by which self-compassion may impact outcomes in sickle cell disease warrant further investigation. Our study contributes to understanding the psychological and social dimensions of the biopsychosocial model. By examining self-compassion in relation to pain, stigma, and psychological well-being, our findings highlight the importance of addressing emotional resilience and social connections in holistic care for individuals living with sickle cell disease. This underscores the value of integrating psychological and social interventions into biomedical approaches, aligning with the biopsychosocial framework to enhance overall well-being.

Future study can consider an interventional study and longitudinal study to assess the impact of self-compassion on chronic pain among people living with sickle cell disease. Additionally, exploring the influence of cultural factors, such as Ghanaian collectivist culture, on shaping self-compassion and its effects could provide valuable insights.
