# Supplementary material for: Does self-compassion moderate the relationship between stigma, pain and psychological well-being among people living with sickle cell disease in Ghana?
Source: PLOS Ment Health. 2026 Feb 23;3(2):e0000556. doi: 10.1371/journal.pmen.0000556 (PMC12928473; doi:10.1371/journal.pmen.0000556)
Supplement: Attachment — Submitted filename: Rebuttal letter_ response reviewer_dna_ 05052025.docx [file pmen.0000556.s003.docx]

**Respond to Editor**

| **Comment** | **Author Response** |
| --- | --- |
| 1. (i,ii,iii,iv) Please clarify all sources of funding (financial or material support) for your study. List the grants (with grant number) or organizations (with url) that supported your study, including funding received from your institution. | We provided the source of finance under ***Financial Disclosure*** after the ***Acknowledgement*** in the document, which we have highlighted in Red for your reference. |
| 2. Please make sure the funding information on the submission form matches your financial disclosure statement. Please indicate by return the full and correct funding information for your study and confirm the order in which funding contributions should appear. Please be sure to indicate whether the funders played any role in the study design, data collection and analysis, decision to publish, or preparation of the manuscript. | This information has also been provided under the ***Financial Disclosure section***. |
| 3. We have amended your Competing Interest statement to comply with journal style. We kindly ask that you double check the statement and let us know if anything is incorrect. | You provided that the ***Competing Interest statement*** has been amended to comply with journal style, however, we do not have access to the current ***competing interest statement*** you provided. |
| 4. Please send separate figure files in .tif or .eps format. Also, remove the figures from your manuscript file but keep the legends | We have moved the figure from the manuscript and provided it in a separate figure file, which we have attached to the submission form as eps format. |
| 5. We have noted that ETHICAL CLEARANCE file is uploaded as file type of 'Supporting Information'. Kindly change to ‘Other’ | We uploaded the ethical clearance as ‘Other’ instead of ‘Supporting Information’ [ECH 334_21-22] |
| 6. In the online submission form, you indicated that “The data is available upon request from the first author. However, since the data can be easily identified because participants are people living with sickle cell conditions and were sampled from an NGO at Korle-Bu Teaching Hospital, they can easily be traced. Therefore, the data is not placed in any public database but can be made available upon specific request.” | We have changed the data availability statement. The current statement which is also under Statements and Declarations read, “*The data underlying this study are not publicly available in order to protect the privacy and confidentiality of participants, as they can be easily identified due to their specific characteristics and the context of the study. However, data can be made available by the first author upon reasonable request, provided that appropriate measures are taken to ensure participant anonymity and ethical standards are upheld”.* This is highlighted in red and can also be found on page 18. |
|  | In your current email you raised a concern of 138 not being enough sample size for a quantitative study, however, we justified this by the sensitivity analysis we conducted which suggests a sample size of 138 . Kindly check on page 7 on the revised manuscript with track changes. Additionally, you stated in your email that we did not justify why we selected our sample from NGOs, on page 6 under the procedure section, we justified that. |

**Respond to Reviewer # 1**

| **Comment** | **Author Response** |
| --- | --- |
| **Methodology section** | |
| 1. Mention whether this study is quantitative or qualitative in this section. | The reviewer suggested that we mention if it is a quantitative or qualitative study under the methodology section of which we have provided that under the study design on page 4 and highlighted in red. |
| 2. Why did you choose two sampling techniques? Why not one? | We employed both purposive and convenience sampling methods to ensure a practical and effective recruitment process. Purposive sampling was used to specifically select individuals living with sickle cell disease who were actively participating in NGO meetings. Additionally, convenience sampling was applied to include those who were readily available during the organization's meetings, as this timing aligned with our data collection process. Further details on the sampling approach are provided in the Procedure section |
| 3. Elaborate on techniques for selecting participants. Mention the method and formula for it, if any. Also, on what basis were non-governmental organizations chosen for this study? | We specifically sampled individuals from NGOs because people living with sickle cell disease often face stigma from healthcare professionals during crises, who may label them as drug-seeking (Bulgin et al., 2018; Jenerette et al., 2015). This stigma can negatively impact their mental health. However, research suggests that individuals who receive social support are more likely to cope effectively during crises (Li et al., 2021; Ozbay et al., 2007). Therefore, selecting participants from NGOs that provide social support, advocacy, and education enabled us to better explore the relationships between self-compassion, pain, stigma, and well-being in this population. *This explanation has been given and added under the procedure section. On the basis of elaborating on the sampling technique, we provided information under the study design and also further elaboration on how we sampled them in the procedure section . All can be found on page 6* |
| 4. Mention recruited but participated separately in this section. | The comment about ***recruited but participated separately in this section*** is not very clear to me. |
| 5. How was the sample size calculated? How can you ensure the sample size of this study will be generalized? | We have added this “ *The sample size was determined using the rule of thumb suggested by Tabachnick and Fidell (2013), which recommends that the sample size (n) for regression analysis should be equal to or exceed 50 + 8m, where 'm' is the number of predictor variables. In this study, with three predictors (stigma, self-compassion, and pain), the minimum required sample size was 74. A sample size of 138 was considered statistically adequate, exceeding the minimum requirement to justify our sample size determination* under ***Participants*** section and it is highlighted in ***red*** and can be found on **page 4** |
| 6. Also, data analysis should be clear. Mention which test was used in the study. | Thank you for your feedback on this. As outlined in the ***Data Analysis section,*** *we detailed the exact analyses conducted in our study.* Specifically, we mentioned that we used the **PROCESS MACRO in SPSS** to perform the moderation analysis, which tested for the interaction effects of our study variables as well as initially using the correlations to examine the relationships between our study variables. We have highlighted them in red for ease of reference and can be found on **page 7.** |
| 7. Overall, this section is unclear. I would suggest dividing the information into sub-headings. For example: Study setting, study design, tools and techniques and so on) | Thanks for the feedback on dividing the study into subsections, We actually have the method divided into sections, we have the ***study design section, participants, measures , procedures and data analysis***. Under these sections we provided detailed information about the design we used, the total number of people we selected, how we arrived at the total number of participants, the selection procedure and the analytical tools used for our analysis. |
| **Comments and Suggestions** | |
| Even though the paper is informative, it still needs a lot of improvement.  • Follow the manuscript guidelines precisely rather than the report format.  • All of the sections need a major revision as the content can be made concise and to the point.  • There are also some spelling errors, line spacing issues, syntax errors, grammar, etc  • Add abbreviations and full forms properly.  • Citations should be mentioned for the scales used in the content, not in the title.  • Don’t mix up the content of other sections. (example: don’t mention the findings in the intro section, no findings in methodology section, etc).  • Format should be followed. The information should be short and precise without losing the current insights. | We have improved on the paper and made any corrections in respect to the grammatical or spelling errors.For instance, on page 1, we closed some spaces in paragraph 2, sentence 3. Also, we changed some sentences to make it more concise. On page 5, we rewrote the sentence to make it concise; see here, *The psychological well-being scale (33-34) was used to measure psychological well-being. It consists of 18 items which assess the well-being of a person. Responses range from 1 (strongly agree) to 7 (strongly disagree), with some items reversed scored. The Cronbach’s alpha for our sample was .71. See Table 2 below.* Citations for the scales have been moved into the content. Abbreviations and full forms properly provided. **Check from page 5 to 6 and highlight them in red as well.** |

**Respond to Reviewer 2**

| **Comment** | **Author Response** |
| --- | --- |
| 1. **Abstact** | |
| a. Instead of writing "Sickle cell disease (SCD) is an inherited genetic disorder that affects hemoglobin." You could write that "Sickle cell disease (SCD), a chronic genetic disorder, is associated with severe pain, stigma, and psychological distress, particularly in low-resource settings like Ghana." | Thanks for your feedback. The wording has been changed |
| b. When describing the sample of your study you should mention the demographic context and the Mean and Standard deviation values for example: "We recruited 138 adults (46 [33.3%] males, 92 [66.7%] females; mean age = 30.99, SD = 8.44) with SCD from two NGOs in Accra, Ghana." | Thank you for your feedback regarding the demographic context, mean, and standard deviation. We modified it to capture your suggestions. |
| c. Instead of "We found that self-compassion had an inverse relationship with both pain and stigma" which lacks statistical clarity, you should write "Self-compassion demonstrated significant negative correlations with pain intensity (r = -.21, p < .01) and stigma (r = -.37, p < .01). | Thank you for your comment. We have modified the write-up to state that “self-compassion had significant negative correlations with pain and stigma |
| d. Readability could be enhanced for this sentence: “However, we did not find a moderating relationship between pain and psychological well-being, nor between stigma and psychological well-being”. It could be rephrased as: "Self-compassion did not moderate the effects of pain (B = .02, p = .512) or stigma (B = -.01, p = .852) on psychological well-being." | The statement has been modified to suit the reviewer’s suggestion, it is highlighted in red. |
| e. “Regardless of this” does not conclude the study findings in an effective manner instead the sentence can be rephrased as "Despite the absence of moderation effects, self-compassion independently predicted better psychological well-being (β = 1.93, p < .001), emphasizing its role as an independent protective factor." | The statement has been modified to suit the suggestion being made by the reviewer and highlighted in red. |
| f. Last sentence suggesting implication for future studies is too vague it could be changed to be more directional. | It has been improved and the current version reads like “ Future research should explore the implementation of interventional and longitudinal studies focused on enhancing self-compassion to examine its causal effects on pain management, stigma reduction, and improvements in psychological well-being among individuals living with sickle cell disease”. |
| g. The abstract should also insert the study design and essential keywords. | We have included the study design in the second sentence in the abstract, it is highlighted in red |
| **2. Revisions in Introduction/Literature review:** | |
| a. Explain why psychological interventions (and specifically self-compassion) are needed. | On page 2, we explained why self-compassion and psychological interventions in general are needed for helping people living with sickle cell disease, pain, stigma and well-being. We have highlighted that in red. We built that argument that biomedics have made significant contributions and talked about holistic treatment and psychological interventions complementing medical treatment of sickle cell disease etc, and mentioned self-compassion which has been suggested in the literature as an effective coping mechanism for dealing with stressful life events etc. Kindly refer to page 1 and 2 for this and it is highlighted in red as well. |
| b. Include evidence (if available) on self-compassion in SCD or similar chronic conditions (e.g., sickle cell trait, lupus). If none, explicitly state: "No studies to date have examined self-compassion in SCD populations." | I have explicitly stated that “No studies to date have examined self-compassion in SCD populations to the best of our knowledge since to the best of our knowledge, no study had been published yet on that. We have included this under the ***Current Study section.*** |
| c. No Theoretical Justification. The biopsychosocial model is mentioned but not explicitly tied to self-compassion. | We appreciate the reviewer’s feedback regarding the theoretical justification. To address this, we have clarified in the manuscript that self-compassion aligns with the biopsychosocial model. Specifically, self-compassion involves treating oneself with kindness and understanding, which emphasizes emotional resilience and self-care. These qualities fit within the psychological and social dimensions of the biopsychosocial model, complementing its biological focus. We also cited relevant literature to support self-compassion as an effective coping mechanism for managing the challenges associated with sickle cell disease, such as pain, stigma, and emotional distress. This addition has been incorporated on pages 1 and 2 and highlighted for your reference. |
| d. The Ghana/Africa focus is underemphasized in the literature review (most cited studies are global). Highlight the unique cultural and systemic challenges in Ghana (e.g., limited healthcare access, societal stigma) to justify the study’s regional relevance. | We appreciate the reviewer’s thoughtful feedback on the need to emphasize the Ghana/Africa context. We cited literature on sickle cell disease in Ghana and Africa on pages 1 under the ***introduction*** and 3, under the ***Current Study section***. These have been highlighted for your reference. |
| e. Self-compassion’s direct effects are reported, but pathways (e.g., via reduced catastrophizing) are unexplored. Explore indirect pathways (e.g., self-compassion → reduced catastrophizing → lower pain). | We thank the reviewer for the insightful feedback. We acknowledge the importance of exploring potential pathways, such as self-compassion’s effects via reduced catastrophizing. To further address this point, we have added literature that establishes self-compassion is negatively associated with rumination and psychological symptoms. This suggests that self-compassion may improve psychological health by mitigating the effects of rumination. These additions have been highlighted in the manuscript for your review. |
| **3. Revisions in Results/Discussion Section** | |
| a. In table 3 note two tailed has been written twice (Note. ** p < 0.01 two-tailed, *p < 0.05 two-tailed) | Two-tailed (*<.05) has been deleted |
| b. Explicitly address how selection bias (NGO-based recruitment) and lack of confounder control (e.g., education, employment) may impact generalizability. | We actually acknowledge this as a limitation. Please see page 12. |
| c. Link findings to the biopsychosocial model (e.g., how self-compassion might mediate biological, psychological, and social pathways). | Thank you for this comment. We appreciate the suggestion to link our findings to the biopsychosocial model. However, our analysis focused on moderation rather than mediation, and we did not conduct a mediation analysis to explore pathways (e.g., biological, psychological, and social mechanisms). Furthermore, we have addressed why self-compassion did not moderate pain and stigma as initially hypothesized, which makes it challenging to interpret the findings through the lens of the biopsychosocial model. We have clarified these points in the manuscript and appreciate your thoughtful feedback. |
| d. Resolve minor grammatical errors and citation inconsistencies (e.g “The variance explained in psychological wellbeing (34%)”, “relationship between pain and   psychological well-being among people living sickle cell disease as well as”) | Thank you for this comment. We believe the citation you are referring to regarding the 34% variance corresponds to Table 5, as the results related to this table were presented in the preceding text. To the best of our knowledge, we have also carefully reviewed the manuscript for any minor grammatical errors and corrected them as needed. We appreciate your observation and have made every effort to ensure accuracy and clarity. |
| e. The lack of moderation effects is attributed to "no prior self-compassion training" without empirical support. | Thank you for this observation. We attributed the lack of moderation effects to the absence of prior self-compassion training because participants reported having no prior exposure or training in self-compassion. While this assumption is based on participant self-reports, we recognize that it is not empirically tested within this study. To address this limitation, we have suggested that future research could explore this further through interventional studies to determine whether self-compassion training influences the observed effects. We appreciate the reviewer’s comment and have clarified this point in the manuscript |
| f. No discussion of how Ghanaian cultural norms (e.g., collectivism, stigma perceptions) might influence self-compassion. | Thank you for this thoughtful comment. We appreciate the suggestion to consider the influence of Ghanaian cultural norms, such as collectivism and stigma perceptions, on self-compassion. While this is an important topic, the primary focus of our study was to explore self-compassion as a moderator of pain, stigma, and psychological well-being. As our results indicated that self-compassion did not moderate these variables, we did not extend the discussion to cultural influences. However, we recognize that cultural factors may play a role in shaping self-compassion and its effects, and we suggest that future research could explore these dynamics within the Ghanaian context to enrich the literature. |
| g. Limited linkage to similar studies (e.g., self-compassion in chronic pain outside SCD). Contrast results with self-compassion studies in other chronic illnesses (e.g., diabetes, cancer). | Thank you for this feedback. We have linked our findings to similar studies in the manuscript. For instance, we referenced a study that identified a negative relationship between self-compassion and pain catastrophizing in individuals with persistent musculoskeletal pain. Additionally, we cited another study that found self-compassion was not a significant independent predictor of pain intensity. These references have been highlighted in red for your convenience. We hope these linkages address the concern and provide the necessary context to situate our findings within existing literature. |
| h. Neurobiological mechanisms (e.g., serotonin/oxytocin) are mentioned but not directly measured or tested. | Thank you for this comment. We acknowledged the neurobiological mechanisms underlying psychological interventions like self-compassion by citing relevant studies to explain how they contribute to regulating the nervous system (e.g., through the influence of serotonin and oxytocin). However, we did not directly measure or test these mechanisms in the current study, as our focus was on the psychological and behavioral aspects of self-compassion. We have clarified this in the manuscript to ensure the scope of our study is clear. |
| 4. **Limitations/conclusion section** | |
| a. Self-report bias (e.g., social desirability in stigma reporting) is not mentioned | We acknowledge the limitation of relying on self-reported data and have included it in the limitation section. It has been highlighted for your reference. |
| b. Lack of control for confounders (e.g., education, SCD genotype) is not acknowledged as a limitation. | Thank you for this comment. We have addressed this comment by providing a sentence in the limitation section. |
| c. Vague calls for "further investigation" without concrete suggestions | Thank you for this feedback. We acknowledge the importance of providing concrete suggestions for future research. In the manuscript, we have suggested further investigation using longitudinal and interventional studies. This recommendation is based on our acknowledgment that, among other factors, self-compassion might not have moderated the relationships in this study due to the lack of prior self-compassion training among participants, as reported. We believe this provides a clear direction for future research to examine whether self-compassion training can influence these outcomes more effectively. |
| d. Conclusions are not tied to the biopsychosocial model introduced earlier, missing an opportunity to frame findings within a holistic care framework. | We added a sentence in the manuscript to address this. It has been highlighted under the conclusion for your reference. |

**Reviewer #3**

| **Comment** | **Author Response** |
| --- | --- |
| This article provides a thorough and well-researched analysis of the Moderating role of self compassion on pain, stigma and psychological well being among people living with sickle cell disease in Ghana . The author does a good job to approach psychological aspects as a holistic approach to help alleviate the suffering of diseased individuals. The writing is clear and easy to follow. The structure of the article is logical, with each section building upon the previous one, making it easy for readers to understand the progression of ideas. This is an informative article that I believe it will contribute well to the field. | Thank you. |
