# Supplementary material for: Does self-compassion moderate the relationship between stigma, pain and psychological well-being among people living with sickle cell disease in Ghana?
Source: PLOS Ment Health. 2026 Feb 23;3(2):e0000556. doi: 10.1371/journal.pmen.0000556 (PMC12928473; doi:10.1371/journal.pmen.0000556)
Supplement: Attachment — Submitted filename: Response to reviewers_10272025.docx [file pmen.0000556.s004.docx]

Author Response Document

| **Abstract** | |
| --- | --- |
| **Reviewers comment** | **Response** |
|  | **We are sincerely grateful to the** anonymous **reviewer**s **for their engagement with our work.** |
| **Reviewer:** **PlosMH SCD article review**  The abstract could be strengthened by specifying the type of stigma examined, as this is relevant for clarifying the hypothesized associations. People with SCD face enacted, perceived, and internalized stigma, and being explicit here would help readers understand the construct under study. In addition, consider presenting the independent effect of self-compassion on psychological well-being before reporting the moderation results, as this would highlight the value of self-compassion itself in addition to its interaction effects | *We have clarified the type of stigma examined by specifying the SCD-specific stigma: social exclusion, internalized stigma, disclosure concerns, and anticipated discrimination.*  *We have incorporated the independent effect of self-compassion on psychological well-being in the abstract, emphasizing its positive association. This effect is reported following the moderation analysis and has been highlighted for ease of review.* |
| **Reviewer: PMEN-D-24-00579_R2** |  |
| The summary provides the essential information (context, method, results and discussion). The only observation to be made pertains to one of the primary outcomes outlined in the abstract. This outcome reveals that self-compassion does not serve to moderate any of the hypothesised effects by the authors (namely stigmatisation or pain on psychological well-being). The title of the article suggests a different result, i.e. that self-compassion does have a moderating effect. In order to circumvent any potential misunderstandings or disillusionment amongst readers, it would be advisable for the authors to consider rephrasing the title in a manner that reflects their primary conclusion.  **Reviewer: Review 3 doc** | *Thank you very much for this thoughtful observation. Our original title was based on the hypothesized moderating role of self-compassion in the relationship between pain, stigma, and psychological well-being. However, as noted, our findings did not support this moderation effect. We appreciate the suggestion to revise the title to better reflect the primary conclusion and avoid potential misinterpretation. We have revised the title of the manuscript to reflect the findings.* |
|  | *We resolved this Reviewer 3’s comment in the previous revisions. As such, the current manuscript reflects the suggestions made by the reviewer.* |
| **Introduction** | |
| **Reviewer:** **PlosMH SCD article review** |  |
| - Instead of quoting the definition of psychosocial well-being in the opening sentence, consider paraphrasing it to maintain a smoother flow. - While the description of self-compassion is strong, it would be helpful to begin with a formal definition and citation before linking it to the biopsychosocial model and prior research. - The introduction could be more complete by including a brief overview of what is already known about psychological well-being among people with SCD. Doing so would clarify how this article contributes to and extends existing work. - Please provide a clear rationale provided for the moderation hypotheses. | - *Thank you for the helpful suggestion. We have revised the opening sentence of the introduction by paraphrasing the definition of hemoglobin to improve flow and maintain consistency in tone. We appreciate your attention to clarity and readability. The part of the sentence has been highlighted in green for easy reference and can be found in page 1.* - *Thank you for this suggestion. We have revised the section on self-compassion to begin with a formal definition and citation from (20-21), which provides conceptual clarity before linking the construct to the biopsychosocial model and prior research. It is highlighted in green on page 2.* - *Thank you for this valuable suggestion. We have expanded the introduction to include a brief overview of existing research on psychological well-being among individuals with SCD. This addition clarifies the relevance of our study and highlights how it extends prior work by focusing on self-compassion in a Ghanaian context. It has been highlighted on page 1 for easy reference.* - *Thank you for highlighting the need for a clearer rationale for our moderation hypotheses. We have revised the "current study" section to include a theoretical justification based on our prior research showing that self-compassion can buffer the psychological effects of pain and stigma. This addition clarifies why we expected moderation and strengthens the conceptual foundation of our hypotheses.* |
| **Reviewer: PMEN-D-24-00579_R2** |  |
| - In Ghana, the disease affects roughly 15,000 newborn babies (3), also, it has been established that one in fifty births are affected, that translates into about 14,000 babies being affected by the disease (6). Out of 2,400 babies born in Ghana on the New Year’s Day in 2018, a small percentage were born with sickle cell disease, unbeknownst to their parents (6).’   It is recommended that the authors consider revising the final sentences of the first paragraph in order to enhance the clarity and conciseness of the information communicated. The final sentence in particular is confusing and requires further elucidation.   - I would urge the authors to present the symptoms of sickle cell disease more precisely. The current formulations are vague and do not convey an in-depth understanding of the disease. Sickle cell disease is characterised in particular by a triad of symptoms: chronic haemolytic anaemia, recurrent vaso-occlusive crises (VOC), progressive organ damage leading to a reduction in life expectancy (Houwing et al., 2019). From a psychological standpoint, this triad also has consequences, including a diminished quality of life (Panepinto et al., 2019), a reduced psychological well-being (Pecker & Darbari, 2019), and feelings of stigmatisation (Ezenwa et al., 2016). - It is recommended that the authors adopt a more precise approach in their presentation of the treatments. In addition to treatments such as bone marrow transplants or gene therapy (whose confidentiality is paramount), there is a long-terme treatment (hydroxycarbamide) which, although not an analgesic, helps to reduce the frequency of CVO (Shah et al., 2019). In the event of CVO, the recommended treatment is opioid (Booker et al., 2006), with the associated consequences (Ruta & Ballas, 2016). Non-pharmaceutical treatments are underexplored (Anie & Green, 2015; E. Chen et al., 2004; L. Y. Edwards & Edwards, 2010; Hildenbrand et al., 2014), and the extent of their efficacy remains a subject of debate (H. Williams & Tanabe, 2016). - Information repeated in the first and fourth sentences. - In your final paragraph, you should state your research objectives without giving any results | - *Thank you for pointing out the need for clarity. We have revised the final sentences of the first paragraph to improve conciseness and enhance the precision of the prevalence data. We highlighted that in green on page 1 for easy reference.* - *We appreciate the recommendation to enhance the clinical precision of our symptom description. We have revised the paragraph to reflect the triad of symptoms and their psychological implications, incorporating the suggested citations.* - *Thank you very much for this thoughtful feedback. We appreciate the suggestion to elaborate on treatment approaches. However, our intention in this section was not to provide a comprehensive overview of clinical treatments for SCD. Rather, we aimed to highlight that most pain management strategies are biomedical in nature, yet many individuals continue to experience distress. This underscores the need to explore complementary psychological interventions that may support pain coping and emotional well-being. As such, our focus is on the psychosocial dimensions of care rather than detailing treatment protocols.* - *Thank you for this comment. We have removed information to avoid redundancy.* - *Thank you for this suggestion, we removed the results from there.* |
|  |  |
| Reviewer: review 3doc |  |
| a) Include evidence (if available) on self-compassion in SCD or similar chronic conditions (e.g., sickle cell trait, lupus). If none, explicitly state: "No studies to date have examined self-compassion in SCD populations."  b) No Theoretical Justification. The biopsychosocial model is mentioned but not explicitly tied to self-compassion.  c) The Ghana/Africa focus is underemphasized in the literature review (most cited studies are global). Highlight the unique cultural and systemic challenges in Ghana (e.g., limited healthcare access, societal stigma) to justify the study’s regional relevance.  d) Self-compassion’s direct effects are reported, but pathways (e.g., via reduced catastrophizing) are unexplored. Explore indirect pathways (e.g., self-compassion → reduced catastrophizing → lower pain). | All these corrections were done in the previous review. We have highlighted those in red for easy reference (check on page 1 and 2).  It seems the Reviewer did not have the revised manuscript. Kindly check the revised manuscript as all the comments have been previously addressed. |
| **Methodology** | |
| **Reviewer:** **PlosMH SCD article review** |  |
| - Suggest placing all information about sampling and recruitment before describing measures. | - *Thank you for this suggestion. We have revised the structure of the Method section by placing all information about sampling and recruitment (Procedure) before the Measure section, as recommended.* |
| - *For recruitment, add more about how participants were identified and contacted within the NGOs and clarify what is meant by the combination of convenience and purposive sampling. Related, it is interesting that authors chose to sample from NGOs because these participants may be coping better. This rationale is not clear, nor is the rationale for not recruiting at healthcare sites; the presence of stigma does not seem to point to a rationale for not including those individuals who may represent those most in need of future interventions. Authors should ensure the limitations of this narrower sample is acknowledged in Discussion.* | - Thank you. We have added information about recruitment and we have explained what convenience and purposive means here. Also, we have acknowledged the recruitment strategy as a limitation under the limitation section. |
| - *Consider separating recruitment/sampling procedures from data collection procedures, perhaps with subheadings for clarity.* | Thank you for this suggestion. We have revised the Procedure section to clearly separate recruitment/sampling from data collection using subheadings for clarity |
| - *For data collection, suggest making the text more concise and providing specific numbers per data collection method. Please also specify whether in-person data collection was interviewer-administered or paper/pencil.* | In the Data Collection subsection, we now specify the number of participants per method (in-person, online, and phone-administered), and we clarify that in-person data collection was self-administered using paper-and pencil questionnaires. We also streamlined the description to improve readability and align with journal expectations. |
| - *Please cite the sample size rule of thumb.* | We did cite the sample size rule but because the referencing style of the journal requires to use numbers, which was referenced as (31) and in the main references is number 31, Tabanick et al. See page 4, it has been highlighted in green for easy reference. |
| - *Demographics might be better placed in the Results, while the Methods could focus on data collected and sampling methods.* | We have moved the demographic table to the result section. |
| - *Please clarify education levels (e.g., what is meant by basic, tertiary) since these terms are not be familiar across contexts.* | In the Ghanaian context basic school is the primary education , secondary is high school and tertiary is college levels. We have explained that under Table `1, and highlighted that in green too for easy reference. |
| - *The Data Analysis section requires more clarity and detail, including explicit explanation of the variables, categories, and analytic steps, and connecting these methods directly to the hypotheses.* | We have addressed this and highlighted them in green in the data analytic section. |
| - *For all measures, describe whether they have been used in this setting, whether they were adapted, and whether they were translated, with detail on those processes.* | With the exception of the self-compassion scale, all the other measures have been used in different population with good reliability and validity. |
| ***Results*** | |
| **Reviewer:** **PlosMH SCD article review** |  |
| - There are missing words and typos that should be corrected. | *We have reviewed and corrected typos and missing words in this section.* |
| - The opening paragraph currently includes additional details about analysis that belong in the Methods section. | *Thank you for bringing this up. We understand your concern. However, in response to a previous reviewer's suggestion, we included a brief overview of what we did to help orient readers. Our intention was not to present full methodological details, but rather to provide a concise summary that reinforces the study's relevance. That said, we are open to revising the paragraph further if the editorial team prefers a more streamlined introduction here.* |
| - Results should be organized by hypothesis, using the sequence in the Abstract as a possible guide. The reader should be able to match research questions and hypotheses (from the Introduction) with the corresponding methods and analyses, and then with the results, in a straightforward and linear fashion. | *Thank you for this helpful suggestion. Our results were organized to reflect the study’s hypotheses, with Table 3 presenting the correlational predictions and Table 4 addressing the moderation analyses. Due to the journal’s word limit of 4,000 words, we opted to combine related results rather than segmenting the section by individual hypotheses. This approach allowed us to present the findings concisely while maintaining alignment with the research questions. However, we are open to further restructuring if the editorial team prefers a more explicitly labeled format.* |
| - Please consult resources related to standards for reporting moderation results and follow these closely in revising. | *Thank you for this helpful suggestion. We consulted APA guidelines and followed recommended standards for reporting moderation analyses. However, if there is a preferred reporting format or structure you would like us to adopt, we would be happy to consider it and make adjustments accordingly.* |
| - Descriptive results on psychological well-being, levels of self-compassion, and levels of stigma in this population should be carefully described, as this is essential for interpreting results. This is especially important as authors mention in the Discussion that perhaps self-compassion was not a moderator because participants had not had a self-compassion intervention. This raises the question about whether the sample perhaps reported very low levels of self-compassion. | *Thank you for this important observation. Descriptive statistics for self-compassion, stigma, pain, and psychological well-being were presented in Table 2 (page 8). In response to your comment, we have strengthened the interpretation of these values in the Discussion section. Specifically, we now highlight that the mean self-compassion score (M = 19.54 out of a possible 60) was relatively low, which may help explain the absence of a moderation effect. This contextual insight supports our argument that future studies should consider self-compassion interventions to assess potential buffering effects more clearly.* |
| **Discussion** | |
| **Reviewer:** **PlosMH SCD article review** |  |
| - Authors should consider alternative explanations for the lack of moderation effects beyond measurement issues or absence of intervention. This argument is currently unclear. More conceptual explanations are needed for why associations may not be present in the hypothesized way. This will be easier once the rationale for the hypotheses is articulated more clearly in the introduction. Authors can then return to that rationale and relevant literature to suggest why expected associations were not observed. | *Thank you for this suggestion. In addition to the methodological considerations we previously discussed, we have expanded the Discussion to include conceptual explanations for the lack of moderation effects. Specifically, we now consider how cultural norms, communal coping strategies, and the chronic nature of SCD pain may influence the role of self-compassion in this population. These additions help clarify why the expected buffering effects may not have emerged and align with the broader literature on psychological adaptation in chronic illness.* |
| - If authors follow the suggestion to report more descriptive results of self-compassion in this population, those findings could be integrated into the discussion. As authors point out, this is one of the first studies of self-compassion in this population, so those results will be valuable. | *Thank you for highlighting the importance of descriptive findings. We have now integrated the descriptive results of self-compassion into the Discussion and reflected on how the relatively low mean score may have influenced the absence of moderation effects.* |
| - The discussion should also highlight the broader contributions of describing psychosocial well-being, stigma, and self-compassion in this under-studied population, even if moderation hypotheses were not supported. | *We appreciate this important reminder. We have revised the final section of the Discussion to emphasize the broader contributions of our findings. Specifically , we highlight how documenting the relationships among pain, stigma, self-compassion, and psychological well-being provides a foundation for future research and intervention development in an under-studied population. These contributions are especially relevant given the limited literature on positive psychological constructs in African health contexts.* |
