# Supplementary material for: Does self-compassion moderate the relationship between stigma, pain and psychological well-being among people living with sickle cell disease in Ghana?
Source: PLOS Ment Health. 2026 Feb 23;3(2):e0000556. doi: 10.1371/journal.pmen.0000556 (PMC12928473; doi:10.1371/journal.pmen.0000556)
Supplement: Peer review history

# Peer review: Decision Letter 0

**DOI:** 10.1371/journal.pmen.0000556.r001

**Authors:** Jinjin Lu

18 Mar 2025

PMEN-D-24-00579

The moderating role of self-compassion on pain, stigma and psychological well-being among people living with Sickle cell disease in Ghana.

PLOS Mental Health

Dear Dr. Agyei,

Thank you for submitting your manuscript to PLOS Mental Health. After careful consideration, we feel that it has merit but does not fully meet PLOS Mental Health’s publication criteria as it currently stands. Therefore, we invite you to submit a revised version of the manuscript that addresses the points raised during the review process.

Please submit your revised manuscript by May 02 2025 11:59PM. If you will need more time than this to complete your revisions, please reply to this message or contact the journal office at mentalhealth@plos.org. When you're ready to submit your revision, log on to https://www.editorialmanager.com/pmen/ and select the 'Submissions Needing Revision' folder to locate your manuscript file.

Please include the following items when submitting your revised manuscript:

- A rebuttal letter that responds to each point raised by the editor and reviewer(s). You should upload this letter as a separate file labeled 'Response to Reviewers'.
- A marked-up copy of your manuscript that highlights changes made to the original version. You should upload this as a separate file labeled 'Revised Manuscript with Track Changes'.
- An unmarked version of your revised paper without tracked changes. You should upload this as a separate file labeled 'Manuscript'.

Guidelines for resubmitting your figure files are available below the reviewer comments at the end of this letter.

We look forward to receiving your revised manuscript.

Kind regards,

Jinjin Lu, Ph.D.

Academic Editor

PLOS Mental Health

Journal Requirements:

1. Please amend your detailed Financial Disclosure statement. This is published with the article. It must therefore be completed in full sentences and contain the exact wording you wish to be published.

i. Please clarify all sources of funding (financial or material support) for your study. List the grants (with grant number) or organizations (with url) that supported your study, including funding received from your institution.

ii. State the initials, alongside each funding source, of each author to receive each grant.

iii. State what role the funders took in the study. If the funders had no role in your study, please state: “The funders had no role in study design, data collection and analysis, decision to publish, or preparation of the manuscript.”

iv. If any authors received a salary from any of your funders, please state which authors and which funders.

2. Please make sure the funding information on the submission form matches your financial disclosure statement. Please indicate by return the full and correct funding information for your study and confirm the order in which funding contributions should appear. Please be sure to indicate whether the funders played any role in the study design, data collection and analysis, decision to publish, or preparation of the manuscript.

3. We have amended your Competing Interest statement to comply with journal style. We kindly ask that you double check the statement and let us know if anything is incorrect.

4. Please send separate figure files in .tif or .eps format. Also, remove the figures from your manuscript file but keep the legends.

For more information about figure files please see our guidelines:

https://journals.plos.org/mentalhealth/s/figures

https://journals.plos.org/mentalhealth/s/figures#loc-file-requirements

5. We have noted that ETHICAL CLEARANCE file is uploaded as file type of 'Supporting Information'. Kindly change to ‘Other’.

6. In the online submission form, you indicated that “The data is available upon request from the first author. However, since the data can be easily identified because participants are people living with sickle cell conditions and were sampled from an NGO at Korle-Bu Teaching Hospital, they can easily be traced. Therefore, the data is not placed in any public database but can be made available upon specific request.”

All PLOS journals now require all data underlying the findings described in their manuscript to be freely available to other researchers, either

1. In a public repository,

2. Within the manuscript itself, or

3. Uploaded as supplementary information.

This policy applies to all data except where public deposition would breach compliance with the protocol approved by your research ethics board. If your data cannot be made publicly available for ethical or legal reasons (e.g., public availability would compromise patient privacy), please explain your reasons by return email and your exemption request will be escalated to the editor for approval. Your exemption request will be handled independently and will not hold up the peer review process, but will need to be resolved should your manuscript be accepted for publication. One of the Editorial team will then be in touch if there are any issues.

Please review your reference list to ensure that it is complete and correct. If you have cited papers that have been retracted, please include the rationale for doing so in the manuscript text, or remove these references and replace them with relevant current references. Any changes to the reference list should be mentioned in the rebuttal letter that accompanies your revised manuscript. If you need to cite a retracted article, indicate the article’s retracted status in the References list and also include a citation and full reference for the retraction notice.

Additional Editor Comments (if provided):

[Note: HTML markup is below. Please do not edit.]

Reviewers' comments:

Reviewer's Responses to Questions

**Comments to the Author**

1. Does this manuscript meet PLOS Mental Health’s publication criteria?

Reviewer #1: No

Reviewer #2: Yes

Reviewer #3: Yes

**********

2. Has the statistical analysis been performed appropriately and rigorously?

Reviewer #1: I don't know

Reviewer #2: Yes

Reviewer #3: Yes

**********

3. Have the authors made all data underlying the findings in their manuscript fully available (please refer to the Data Availability Statement at the start of the manuscript PDF file)?

The PLOS Data policy

Reviewer #1: Yes

Reviewer #2: No

Reviewer #3: Yes

**********

4. Is the manuscript presented in an intelligible fashion and written in standard English?

Reviewer #1: Yes

Reviewer #2: Yes

Reviewer #3: Yes

**********

Reviewer #1: This study is interesting enough as many variables connect with self-compassion in people who have the mentioned disease. Although this study has potential, unfortunately, in my opinion, it doesn’t meet any of the criteria of the manuscript writing. Firstly, there should be line numbers so reviewers can easily address the lines you should work on. Others, I have added some suggestions below as well.

Queries

Methodology section

• Mention whether this study is quantitative or qualitative in this section.

• Why did you choose two sampling techniques? Why not one?

• Elaborate on techniques for selecting participants. Mention the method and formula for it, if any. Also, on what basis were non-governmental organizations chosen for this study?

• Mention recruited but participated separately in this section.

• How was the sample size calculated? How can you ensure the sample size of this study will be generalized?

• Also, data analysis should be clear. Mention which test was used in the study.

• Overall, this section is unclear. I would suggest dividing the information into sub-headings. For example: Study setting, study design, tools and techniques and so on)

Comments and Suggestions

• Even though the paper is informative, it still needs a lot of improvement.

• Follow the manuscript guidelines precisely rather than the report format.

• All of the sections need a major revision as the content can be made concise and to the point.

• There are also some spelling errors, line spacing issues, syntax errors, grammar, etc

• Add abbreviations and full forms properly.

• Citations should be mentioned for the scales used in the content, not in the title.

• Don’t mix up the content of other sections. (example: don’t mention the findings in the intro section, no findings in methodology section, etc).

• Format should be followed. The information should be short and precise without losing the current insights.

Reviewer #2: 1. The wording and sentence structures in the abstract of the research article could be refined to enhance clarity, coherence, and readability for example:

a) Instead of writing "Sickle cell disease (SCD) is an inherited genetic disorder that affects hemoglobin." You could write that "Sickle cell disease (SCD), a chronic genetic disorder, is associated with severe pain, stigma, and psychological distress, particularly in low-resource settings like Ghana."

b) When describing the sample of your study you should mention the demographic context and the Mean and Standard deviation values for example: "We recruited 138 adults (46 [33.3%] males, 92 [66.7%] females; mean age = 30.99, SD = 8.44) with SCD from two NGOs in Accra, Ghana."

c) Instead of "We found that self-compassion had an inverse relationship with both pain and stigma" which lacks statistical clarity, you should write "Self-compassion demonstrated significant negative correlations with pain intensity (r = -.21, p < .01) and stigma (r = -.37, p < .01).

d) Readability could be enhanced for this sentence: “However, we did not find a moderating relationship between pain and psychological well-being, nor between stigma and psychological well-being”. It could be rephrased as: "Self-compassion did not moderate the effects of pain (B = .02, p = .512) or stigma (B = -.01, p = .852) on psychological well-being."

e) “Regardless of this” does not conclude the study findings in an effective manner instead the sentence can be rephrased as "Despite the absence of moderation effects, self-compassion independently predicted better psychological well-being (β = 1.93, p < .001), emphasizing its role as an independent protective factor."

f) Last sentence suggesting implication for future studies is too vague it could be changed to be more directional.

g) The abstract should also insert the study design and essential keywords.

2. Revisions in Introduction/Literature review:

a) Explain why psychological interventions (and specifically self-compassion) are needed.

b) Include evidence (if available) on self-compassion in SCD or similar chronic conditions (e.g., sickle cell trait, lupus). If none, explicitly state: "No studies to date have examined self-compassion in SCD populations."

c) No Theoretical Justification. The biopsychosocial model is mentioned but not explicitly tied to self-compassion.

d) The Ghana/Africa focus is underemphasized in the literature review (most cited studies are global). Highlight the unique cultural and systemic challenges in Ghana (e.g., limited healthcare access, societal stigma) to justify the study’s regional relevance.

e) Self-compassion’s direct effects are reported, but pathways (e.g., via reduced catastrophizing) are unexplored. Explore indirect pathways (e.g., self-compassion → reduced catastrophizing → lower pain).

3. Revisions in Results/Discussion Section

a) In table 3 note two tailed has been written twice (Note. ** p < 0.01 two-tailed, *p < 0.05 two-tailed)

b) Explicitly address how selection bias (NGO-based recruitment) and lack of confounder control (e.g., education, employment) may impact generalizability.

c) Link findings to the biopsychosocial model (e.g., how self-compassion might mediate biological, psychological, and social pathways).

d) Resolve minor grammatical errors and citation inconsistencies (e.g “The variance explained in psychological wellbeing (34%)”, “relationship between pain and

psychological well-being among people living sickle cell disease as well as”)

e) The lack of moderation effects is attributed to "no prior self-compassion training" without empirical support.

f) No discussion of how Ghanaian cultural norms (e.g., collectivism, stigma perceptions) might influence self-compassion.

g) Limited linkage to similar studies (e.g., self-compassion in chronic pain outside SCD). Contrast results with self-compassion studies in other chronic illnesses (e.g., diabetes, cancer).

h) Neurobiological mechanisms (e.g., serotonin/oxytocin) are mentioned but not directly measured or tested.

4. Limitations/Conclusion section

a) Self-report bias (e.g., social desirability in stigma reporting) is not mentioned

b) Lack of control for confounders (e.g., education, SCD genotype) is not acknowledged as a limitation.

c) Vague calls for "further investigation" without concrete suggestions

d) Conclusions are not tied to the biopsychosocial model introduced earlier, missing an opportunity to frame findings within a holistic care framework.

Reviewer #3: This article provides a thorough and well-researched analysis of the Moderating role of self compassion on pain , stigma and psychological well being among people living with sickle cell disease in Ghana . The author does a good job to approach psychological aspects as a wholistic approach to help alleviate the suffering of diseased individuals.

The writing is clear and easy to follow. The structure of the article is logical, with each section building upon the previous one, making it easy for readers to understand the progression of ideas. This is an informative article that I believe it will contribute well to the field.

**********

what does this mean? ). If published, this will include your full peer review and any attached files.

**Do you want your identity to be public for this peer review?** If you choose “no”, your identity will remain anonymous but your review may still be made public.

For information about this choice, including consent withdrawal, please see our Privacy Policy

Reviewer #1: No

Reviewer #2: **Yes:** Tayyaba Ali

Reviewer #3: No

**********

[NOTE: If reviewer comments were submitted as an attachment file, they will be attached to this email and accessible via the submission site. Please log into your account, locate the manuscript record, and check for the action link "View Attachments". If this link does not appear, there are no attachment files.]

While revising your submission, please upload your figure files to the Preflight Analysis and Conversion Engine (PACE) digital diagnostic tool, https://pacev2.apexcovantage.com/. PACE helps ensure that figures meet PLOS requirements. To use PACE, you must first register as a user. Registration is free. Then, login and navigate to the UPLOAD tab, where you will find detailed instructions on how to use the tool. If you encounter any issues or have any questions when using PACE, please email PLOS at figures@plos.org. Please note that Supporting Information files do not need this step.

# Author comment: Author response to Decision Letter 1

**DOI:** 10.1371/journal.pmen.0000556.r002

11 Apr 2025

Attachment Submitted filename: Rebuttal letter_ response reviewer_dna_.docx

# Editor report: Decision Letter 1

**DOI:** 10.1371/journal.pmen.0000556.r003

**Authors:** Jinjin Lu

30 Apr 2025

PMEN-D-24-00579R1

The moderating role of self-compassion on pain, stigma and psychological well-being among people living with Sickle cell disease in Ghana.

PLOS Mental Health

Dear Dr. Agyei,

Thank you for submitting your manuscript to PLOS Mental Health. After careful consideration, we feel that it has merit but does not fully meet PLOS Mental Health’s publication criteria as it currently stands. Therefore, we invite you to submit a revised version of the manuscript that addresses the points raised during the review process.

Please submit your revised manuscript by May 30 2025 11:59PM. If you will need more time than this to complete your revisions, please reply to this message or contact the journal office at mentalhealth@plos.org. When you're ready to submit your revision, log on to https://www.editorialmanager.com/pmen/ and select the 'Submissions Needing Revision' folder to locate your manuscript file.

Please include the following items when submitting your revised manuscript:

- A rebuttal letter that responds to each point raised by the editor and reviewer(s). You should upload this letter as a separate file labeled 'Response to Reviewers'.
- A marked-up copy of your manuscript that highlights changes made to the original version. You should upload this as a separate file labeled 'Revised Manuscript with Track Changes'.
- An unmarked version of your revised paper without tracked changes. You should upload this as a separate file labeled 'Manuscript'.

Guidelines for resubmitting your figure files are available below the reviewer comments at the end of this letter.

We look forward to receiving your revised manuscript.

Kind regards,

Jinjin Lu, Ph.D.

Academic Editor

PLOS Mental Health

Journal Requirements:

Please review your reference list to ensure that it is complete and correct. If you have cited papers that have been retracted, please include the rationale for doing so in the manuscript text, or remove these references and replace them with relevant current references. Any changes to the reference list should be mentioned in the rebuttal letter that accompanies your revised manuscript. If you need to cite a retracted article, indicate the article’s retracted status in the References list and also include a citation and full reference for the retraction notice.

Additional Editor Comments (if provided):

Please read the feedback provided by the reviewer 2 and 3

[Note: HTML markup is below. Please do not edit.]

Reviewers' comments:

[NOTE: If reviewer comments were submitted as an attachment file, they will be attached to this email and accessible via the submission site. Please log into your account, locate the manuscript record, and check for the action link "View Attachments". If this link does not appear, there are no attachment files.]

While revising your submission, please upload your figure files to the Preflight Analysis and Conversion Engine (PACE) digital diagnostic tool, https://pacev2.apexcovantage.com/. PACE helps ensure that figures meet PLOS requirements. To use PACE, you must first register as a user. Registration is free. Then, login and navigate to the UPLOAD tab, where you will find detailed instructions on how to use the tool. If you encounter any issues or have any questions when using PACE, please email PLOS at figures@plos.org. Please note that Supporting Information files do not need this step.

# Author comment: Author response to Decision Letter 2

**DOI:** 10.1371/journal.pmen.0000556.r004

7 May 2025

Attachment Submitted filename: Rebuttal letter_ response reviewer_dna_ 05052025.docx

# Peer review: Decision Letter 2

**DOI:** 10.1371/journal.pmen.0000556.r005

**Authors:** Steve Zimmerman

10 Sep 2025

PMEN-D-24-00579R2

The moderating role of self-compassion on pain, stigma and psychological well-being among people living with Sickle cell disease in Ghana.

PLOS Mental Health

Dear Dr. Agyei,

Thank you for submitting your manuscript to PLOS Mental Health. After careful consideration, we feel that it has merit but does not fully meet PLOS Mental Health’s publication criteria as it currently stands. Therefore, we invite you to submit a revised version of the manuscript that addresses the points raised during the review process.

The manuscript has been evaluated by three reviewers, and their comments are available below and in the attached files.

Could you please revise the manuscript to carefully address the concerns raised?

Please submit your revised manuscript by Oct 24 2025 11:59PM. If you will need more time than this to complete your revisions, please reply to this message or contact the journal office at mentalhealth@plos.org. When you're ready to submit your revision, log on to https://www.editorialmanager.com/pmen/ and select the 'Submissions Needing Revision' folder to locate your manuscript file.

Please include the following items when submitting your revised manuscript:

- A rebuttal letter that responds to each point raised by the editor and reviewer(s). You should upload this letter as a separate file labeled 'Response to Reviewers'.
- A marked-up copy of your manuscript that highlights changes made to the original version. You should upload this as a separate file labeled 'Revised Manuscript with Track Changes'.
- An unmarked version of your revised paper without tracked changes. You should upload this as a separate file labeled 'Manuscript'.

Guidelines for resubmitting your figure files are available below the reviewer comments at the end of this letter.

We look forward to receiving your revised manuscript.

Kind regards,

Steve Zimmerman, PhD

PLOS Staff Editor

Journal Requirements:

If the reviewer comments include a recommendation to cite specific previously published works, please review and evaluate these publications to determine whether they are relevant and should be cited. There is no requirement to cite these works unless the editor has indicated otherwise.

Additional Editor Comments (if provided):

[Note: HTML markup is below. Please do not edit.]

Reviewers' comments:

Reviewer's Responses to Questions

**Comments to the Author**

Reviewer #4: (No Response)

Reviewer #5: All comments have been addressed

Reviewer #6: (No Response)

**********

publication criteria?

Reviewer #4: Yes

Reviewer #5: Partly

Reviewer #6: Partly

**********

3. Has the statistical analysis been performed appropriately and rigorously?

Reviewer #4: Yes

Reviewer #5: Yes

Reviewer #6: I don't know

**********

4. Have the authors made all data underlying the findings in their manuscript fully available (please refer to the Data Availability Statement at the start of the manuscript PDF file)?

The PLOS Data policy

Reviewer #4: Yes

Reviewer #5: (No Response)

Reviewer #6: (No Response)

**********

5. Is the manuscript presented in an intelligible fashion and written in standard English?

Reviewer #4: Yes

Reviewer #5: No

Reviewer #6: Yes

**********

Reviewer #4: See attached file.

Reviewer #5: This is an important topic for dissemination, however, the manuscript could be improved with an English editor as there are numerous grammatical errors and awkward phrases which distract the reader.

The last sentence of the introduction sentence contains results and should be moved.

Reviewer #6: Please see attached.

**********

what does this mean? ). If published, this will include your full peer review and any attached files.

**Do you want your identity to be public for this peer review?** If you choose “no”, your identity will remain anonymous but your review may still be made public.

For information about this choice, including consent withdrawal, please see our Privacy Policy

Reviewer #4: **Yes:** Damien OUDIN DOGLIONI

Reviewer #5: No

Reviewer #6: No

**********

[NOTE: If reviewer comments were submitted as an attachment file, they will be attached to this email and accessible via the submission site. Please log into your account, locate the manuscript record, and check for the action link "View Attachments". If this link does not appear, there are no attachment files.]

While revising your submission, please upload your figure files to the Preflight Analysis and Conversion Engine (PACE) digital diagnostic tool, https://pacev2.apexcovantage.com/. PACE helps ensure that figures meet PLOS requirements. To use PACE, you must first register as a user. Registration is free. Then, login and navigate to the UPLOAD tab, where you will find detailed instructions on how to use the tool. If you encounter any issues or have any questions when using PACE, please email PLOS at figures@plos.org. Please note that Supporting Information files do not need this step.

# Author comment: Author response to Decision Letter 3

**DOI:** 10.1371/journal.pmen.0000556.r006

27 Oct 2025

Attachment Submitted filename: Response to reviewers_10272025.docx

# Editor report: Decision Letter 3

**DOI:** 10.1371/journal.pmen.0000556.r007

**Authors:** Katherine Kokkinias

27 Jan 2026

The moderating role of self-compassion on pain, stigma and psychological well-being among people living with Sickle cell disease in Ghana.

PMEN-D-24-00579R3

Dear Mr Agyei,

We are pleased to inform you that your manuscript 'The moderating role of self-compassion on pain, stigma and psychological well-being among people living with Sickle cell disease in Ghana.' has been provisionally accepted for publication in PLOS Mental Health.

Before your manuscript can be formally accepted you will need to complete some formatting changes, which you will receive in a follow up email. A member of our team will be in touch with a set of requests.

Please note that your manuscript will not be scheduled for publication until you have made the required changes, so a swift response is appreciated.

IMPORTANT: The editorial review process is now complete. PLOS will only permit corrections to spelling, formatting or significant scientific errors from this point onwards. Requests for major changes, or any which affect the scientific understanding of your work, will cause delays to the publication date of your manuscript.

If your institution or institutions have a press office, please notify them about your upcoming paper to help maximize its impact. If they'll be preparing press materials, please inform our press team as soon as possible -- no later than 48 hours after receiving the formal acceptance. Your manuscript will remain under strict press embargo until 2 pm Eastern Time on the date of publication. For more information, please contact mentalhealth@plos.org.

Thank you again for supporting Open Access publishing; we are looking forward to publishing your work in PLOS Mental Health.

Best regards,

Katherine Demi Kokkinias, Ph.D.

Staff Editor

PLOS Mental Health

***********************************************************

Reviewer Comments (if any, and for reference):
